# LOPDF: a framework for extracting and producing open data of scientific documents for smart digital libraries

**DOI:** 10.7717/peerj-cs.445

**Published:** 2021-04-07

**Authors:** Muhammad Ahtisham Aslam

**Affiliations:** Department of Information Systems, Faculty of Computing and Information Technology, King Abdulaziz University, Jeddah, Makkah, Saudi Arabia

**Keywords:** Digital libraries, Ontological reasoning, Open data, Algorithms analysis

## Abstract

**Background:**

Results of scientific experiments and research work, either conducted by individuals or organizations, are published and shared with scientific community in different types of scientific publications such as books, chapters, journals, articles, reference works and reference works entries. One aspect of these documents is their contents and the other is metadata. Metadata of scientific documents could be used to increase mutual cooperation, find people with common interest and research work, and to find scientific documents in the matching domains. The major issue in getting these benefits from metadata of scientific publications is availability of these data in unstructured (or semi-structured) format so that it can not be used to ask smart queries that can help in computing and performing different types of analysis on scientific publications data. Also, acquisition and smart processing of publications data is a complicated as well as time and resource consuming task.

**Methods:**

To address this problem we have developed a generic framework named as Linked Open Publications Data Framework (LOPDF). The LOPDF framework can be used to crawl, process, extract and produce machine understandable data (i.e., LOD) about scientific publications from different publisher specific sources such as portals, XML export and websites. In this paper we present the architecture, process and algorithm that we developed to process textual publications data and to produce semantically enriched data as RDF datasets (i.e., open data).

**Results:**

The resulting datasets can be used to make smart queries by making use of SPARQL protocol. We also present the quantitative as well as qualitative analysis of our resulting datasets which ultimately can be used to compute the research behavior of organizations in rapidly growing knowledge society. Finally, we present the potential usage of producing and processing such open data of scientific publications and how results of performing smart queries on resulting open datasets can be used to compute the impact and perform different types of analysis on scientific publications data.

## Introduction

In the context of knowledge society, sharing of resources, research results, scientific documents and their metadata over the Web (Taibi et al., 2015) is a key factor enabling mutual collaboration and knowledge sharing. Metadata of scientific documents can help a in the finding of related articles, books, organizations and researchers with common interests, based on the scientific publications. There is a large amount of scientific documents that have already been published and are being organized by large number of publishers by using metadata of these scientific publications for the purpose of data acquisition and processing to compute the individual’s as well as organization’s research behavior and contribution in the rapidly growing knowledge society. When it comes to joint research and common interests, one main limitation of this huge data of scientific documents is that it is publisher specific and not linked with scientific documents published by other publishers such that it can be used to ask smart queries. The main reason of this non collaborative behavior is that the data about scientific documents published by different publishers is not interlinked, available in human understandable format and cannot be acquired and processed by machines as in its current format.

Web browsing shows that most of the publishers (such as *Springer, IEEE, and Elsevier*) provide open access (e.g., *Springer text- and data-mining policy*: http://www.springer.com/gp/rights-permissions/springer-s-text-and-data-mining-policy/29056) to their publications metadata such as document *title, abstract, ISBN, DOI, journal, volume, issue, book, chapter*, through their portal, website or as XML export (Lnenicka, 2015; Unbehauen et al., 2012). Such formats don’t allow distributed linking, machine understanding, acquisition and processing of scientific publications data which ultimately result the huge amount of scientific publications data to be in silence while living in the paradigm of knowledge-based societies.

In addition to this producing machine-understandable and processable data from existing resources (rather than to produce data from scratch) is one of the key challenges for computer science (Koumenides et al., 2010; Hochtl, Reichst & Adter, 2011), (especially in terms of information retrieval (Manning, Raghavan & Schtze, 2008) and feature extraction Dindorf et al., 2020). This topic is becoming more and more important because of its use in creating smart applications and mashups for data acquisition and processing to compute behaviors in knowledge society  (DiFranzo et al., 2011). Information and data extraction algorithms are being improved for better and precise data extraction and linking (Elbassuoni et al., 2010). In the perspective of this work, such algorithms can be used to extract, produce and interlink the data of scientific publications. Here, we refer this interlinked data as Linked Open Scientific Publications Data (LOSPD). This LOSPD is a machine readable description of scientific publications which enables researchers to ask smart questions to find semantically matching scientific documents and researcher for possible research collaboration.

In addition to this, for more than a decade, researchers and practitioners in the domain of semantic technologies have been working on developing different methodologies, frameworks and algorithms to acquire, process, extract and produce LOD from different kind of resources such as relational databases, HTML pages, vendor specific source templates and text documents. For example, different methodologies and frameworks to produce government LOD have been presented in Raamkumar et al. (2015), Sheridan & Tennison (2010) and Liu et al. (2011). Specifically in Raamkumar et al. (2015), the authors present a migrational framework to produce LOD from multi-agencies government sources. Another framework for producing open data from different sources such as XML files, bibtex and CSV files has been presented in Huynh, Karger & Miller (2007). Similarly, a methodology for mapping the plain text of scientific documents to citation typing ontology has been discussed in Krewinkel & Winkler (2017). In addition to this, a Data Integration Framework (DIF) is presented in DIF. The proposed framework can be used for semantic based integration of heterogeneous data sources as well as processes. Similarly, an algorithm and visual tool for extracting and producing geospatial open data is presented in Zhang et al. (2013). The rapid growth in developing frameworks and algorithms to extract and produce LOD from different sources demands the development of a generic frameworks that can be used to produce and link scientific publications metadata with the global LOD cloud so that the large amount of scientific publications metadata can be used to compute and describe the research behavior of different stakeholders in the growing knowledge society by making use of smart queries on the open data.

While understanding the value of publications metadata in machine processable format, in this paper we present the generic framework named the Linked Open Publications Data Framework (LOPDF). The LOPDF framework can be used to produce the LOD from different publisher specific sources by customizing Endpoint triggers of the framework. The resulting datasets can be used to perform different types of research specific analysis on publications data and how results of such analysis can be used in defining organizational research directions. Overall, this paper has the following contributions:

• A generic framework named as Linked Open Publications Data Framework (LOPDF)
• Architecture and data extraction process of the LOPDF framework
• An algorithm that we developed to process the huge amount of scientific publications data from different publisher specific sources
• Quantitative as well as qualitative analysis of extracted datasets to prove the accuracy and performance of the LOPDF framework
• Sample queries and their results through the SPARQL Endpoint (as a proof of qualitative and quantitative aspects of extracted datasets)

## Related Work

With the popularity and beneficial usage of Linked Open Data (LOD), number of organizations publishing their public data as open data and linking it with other datasets are also increasing. Different kinds of frameworks, algorithms and methodologies are being developed and implemented to extract, process and produce LOD from different kind of data sources such as Web pages, CSV files, relational databases and XML files. This growth is resulting in the bigger and bigger LOD cloud which ultimately is resulting in better and bigger knowledge graphs that can be used to apply cognitive computing techniques to describe the behavior of different stakeholders in the research based knowledge society. In this section we present the work related to producing LOD from different kind of existing sources.

A framework (i.e., Exhibit) to produce machine understandable data from different sources is presented in Huynh, Karger & Miller (2007). The Exihibit framework can be used to publish structured data from different sources such as XML files, bibtex, CSV files and Excel sheets. The converted RDF data can be used to create mashups, as input for semantic Web agents and to develop third party applications which need the source data in machine processable format such as RDF. Similarly, a methodology to address the feasibility of writing scientific documents in plain text files and then converting them into common publication formats such as HTML and PDF is described in Krewinkel & Winkler (2017). Further more, how these documents can be translated into citation typing ontology and journal article tag suite is also discussed in this work. The ultimate goal is to minimize the time and cost factors in scientific documents’ formatting. Another framework for cleaning and linking government data from different sources such as HTML pages, Excel sheets and producing RDF data is presented in Knap, Nečaský & Svoboda (2012). The semantically enriched government LOD produced by this framework can be used by citizens to observe and analyze the government performance. The resulting data can also be used by applications to define business policies for the future. The Open Data Clean Store (ODCS) module of the framework plays the key role in cleaning and linking the government data. In addition to this a framework (named as Data Integration Framework (DIF)) is presented in DIF. The proposed framework can be used for semantic based integration of heterogeneous data sources as well as processes. The DIF framework supports to overcome the problems that raise due to semantic heterogeneity of data as well as interoperability issues between different resources by making use of ontologies.

Due to the importance of LOD, different frameworks and methodologies have been developed to produce LOD in different sectors such as government, education and health. Government sector is one which is contributing a huge amount of open data to the global Web of data by publishing and interlinking the government data in the global space. British government, as an example, has published the government LOD (Sheridan & Tennison, 2010) to facilitate citizens to get easy access to their required information in different domains. Australian government is producing and integrating the data (i.e., LOD) from different domains focusing on complex interactions between nature and society (Liu et al., 2011). The Albanian government has taken an initiative to make the government data available (as part of Open Government Partnership) to citizens so that they can participate in governance and decision making as a part of modern democracy. Indonesian government has taken the initiative and is continuously improving its transparency through publishing their government LOD (Aryan et al., 2014). Since, extracting and producing LOD is getting attraction among the researchers and practitioners in different domains therefore, an algorithm to extract, process and integrate geospatial data from different sources is presented in Zhang et al. (2013). In Zhang et al. (2013) authors presented their data retrieval algorithm which in second step is implemented and integrated with visual tool which invoke different services to extract geospatial data from different sources and produces resulting LOD. A joint venture, as an integration of ontologies and blockchain is elaborated in El-dosuky & El-adl (2019). In this work authors describe that how the efficiency and interoperability in e-government services can be improved by applying blockchain and by using ontological reasoning on government data. Additionally, an ontology based assessment framework is presented in Beydoun et al. (2020). The proposed framework makes the use of ontology based approach to identify and reuse the different factors in data oriented framework development. The ontology that is developed and presented in this work can also be used for indexing, representing and reusing the domain concepts.

Similarly, a migrational framework to convert Singapore government data from traditional legacy system to Linked Open Data (LOD) format has been presented in Raamkumar et al. (2015). The proposed framework can be used to produce the open data from multiple government agencies and to link them together so that it could be queried for integrated view of respective datasets. Another framework (i.e., Silk) for transferring structured data sources to RDF datasets is presented in Jentzsch, Isele & Bizer (2010) and Isele, Jentzsch & Bizer (2010). The Silk framework can also be used to find links from publisher specific datasets to other publically available datasets. We have also presented our initial work (Aslam et al., 2016) on extracting and adding semantics to digital libraries. In Aslam et al. (2016) we present the architecture and some basics of LOD extraction algorithm. Our current framework that we presented in this work is much enhanced version of this initial work as our current framework can be used to crawl, identify, extract, process and produce semantically enriched data from different sources such as Web pages, Excel sheets, traditional databases and portals. In addition to this, we have also implemented our current framework to extract publications data from open portals of different scientific publishers such as IEEE and Elsevier and to link them together as Publications Linked Open Data Cloud. The LOPDF framework algorithm has been enhanced and improved sufficiently for better processing and linking of data items. We also customized this framework to produce the local LOD cloud by extracting and linking data in different domains such as government (AlSukhayri et al., 2020) and education (Alrehaili et al., 2014).

## The Integrated Publications Data Source Model

**Figure 1:**
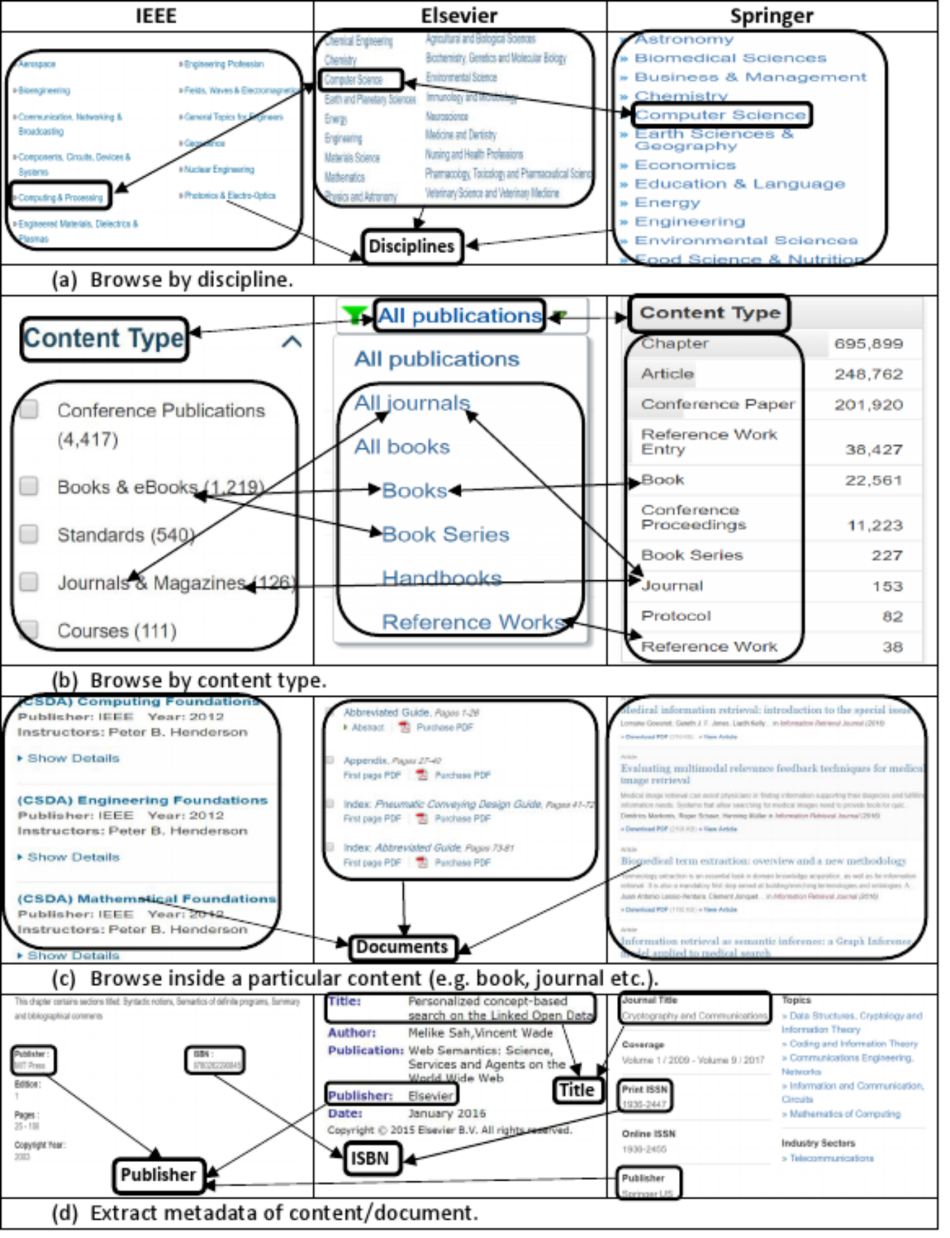
Integrated view of information templates from different publishers showing the (A) list of disciplines, (B) list of content types, (C) list of sub-content types (e.g., chapter, article), (D) and document’s metadata.

The data model of the information source is the most important aspect which needs to be explored carefully to extract and produce accurate data. We explored and investigated the data model of most famous and well-known publishers and came across the following major common structural as well as terminological aspects of the data model: (i) categorization of documents into disciplines such as *computer science, management, engineering* (ii) categorization of documents in to content types such as *book, chapter, journal* (iii) expression of publications metadata by using standard terms such as *title, isbn, publisher*. Figure 1 shows a comparative and integrated data model of some well-known publishers such as *IEEE, Elsevier, Springer*.

The Fig. 1A of the integrated publications source data model shows that for organizational as well as maintenance purposes, publishers categorize scientific documents in disciplines. These disciplines actually refer to the field of research and relation of publications contents to a particular field. Both industry as well as academia can differentiate and categorize scientific contents and contributions to any of these disciplines. This categorization helps other researchers to find scientific contents of their interest and individuals as well as organizations working in particular domain of interest. One important point here is that it is not mandatory that all publishers use the same naming conventions for all disciplines. For example, *computer science* discipline, in IEEE, Elsevier and Springer is referred as *‘Computer Science’, ‘Computing and Processing’ and ‘Computer Science’* respectively. Such kind of name ambiguities in disciplines names are handled by using *owl:sameAs* property. It mean we can define the type of a scientific document based on the discipline in which a document is published (as shown in second statement of Example 1).

A scientific document published in any discipline can either be a book, a chapter of a book, a journal or an article in a journal and so on. This categorization of documents is termed as *content type*. Figure 1B shows the list of *content types* in which documents are categorized by different publishers. Example 1 consists of two RDF statements, the first statement describes that the specified document is a *book chapter* and second statement describes that it is published in *Computer Science* discipline.

Example 1: RDF statements describing a document content type and discipline.

<SPedia:Test_Suites><rdf:type><SPedia:Computer_Science>.

<SPedia:Test_Suites><rdf:type><SPedia:Book_Chapter>.

**Figure 2:**
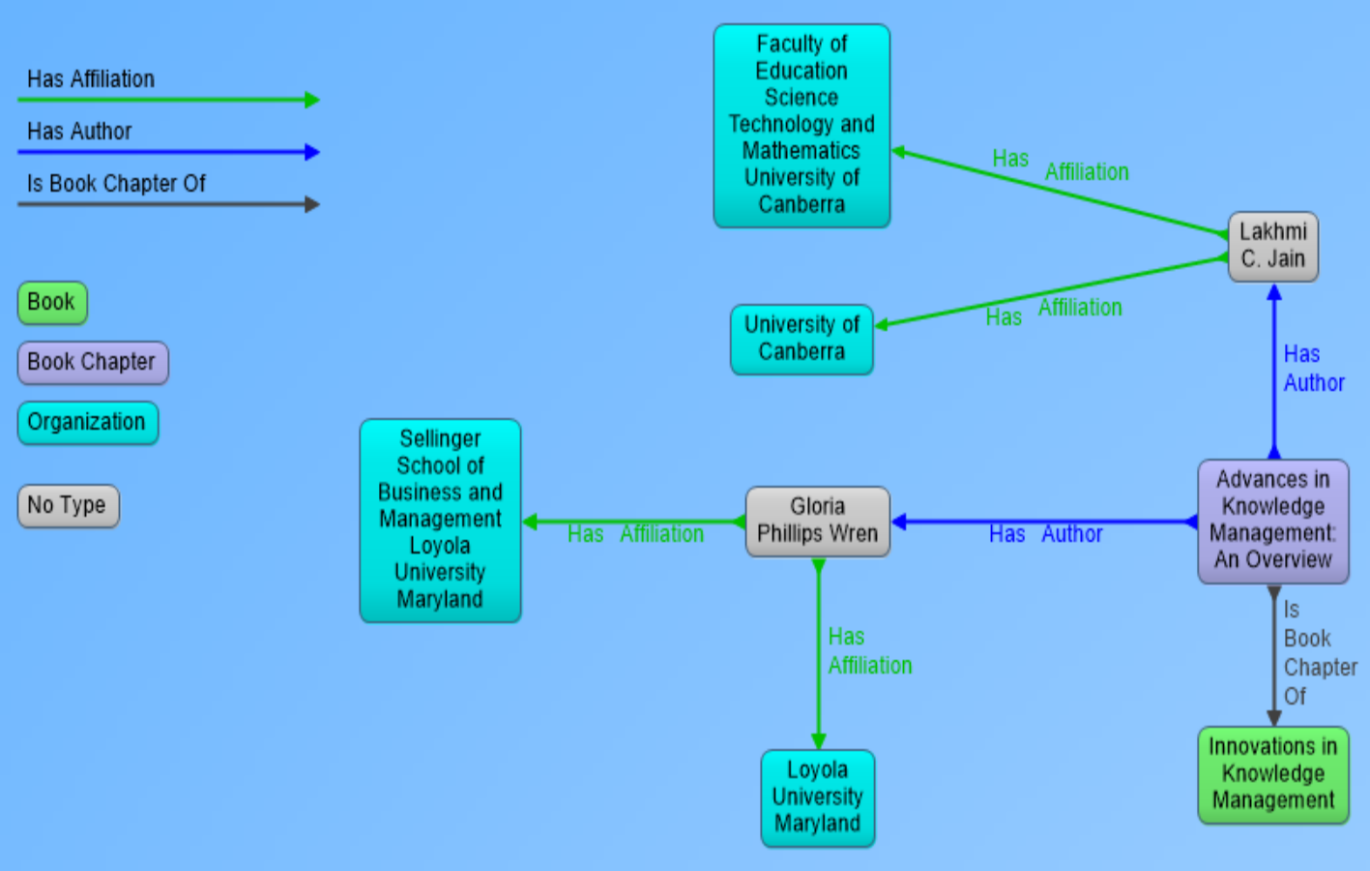
Visual representation of RDF data showing book chapter as a resource and its link to parent book, authors and affiliations of authors.

In the perspective of our research, metadata of scientific documents is most important part of information that needs to be processed and extracted. Metadata of scientific documents plays significant role in linking scientific publications open data with other publically available datasets to create Linked Open Data (LOD). Metadata of scientific documents consist of standard terms such as title, author, organization, doi and publisher (as shown in Fig. 1D. This metadata can be used to establish different links such as the link of a document with its author by using property *has_author*, and author can be linked with his organization by using *has_Affiliation* property (as shown in Fig. 2), and organization can be linked to external dataset (e.g., geonames) by using has_coordinates property and so on. This linking can be used to ask SPARQL based queries to fetch data from multiple interlinked datasets.

## LOPDF Architecture and Data Extraction Process

**Figure 3:**
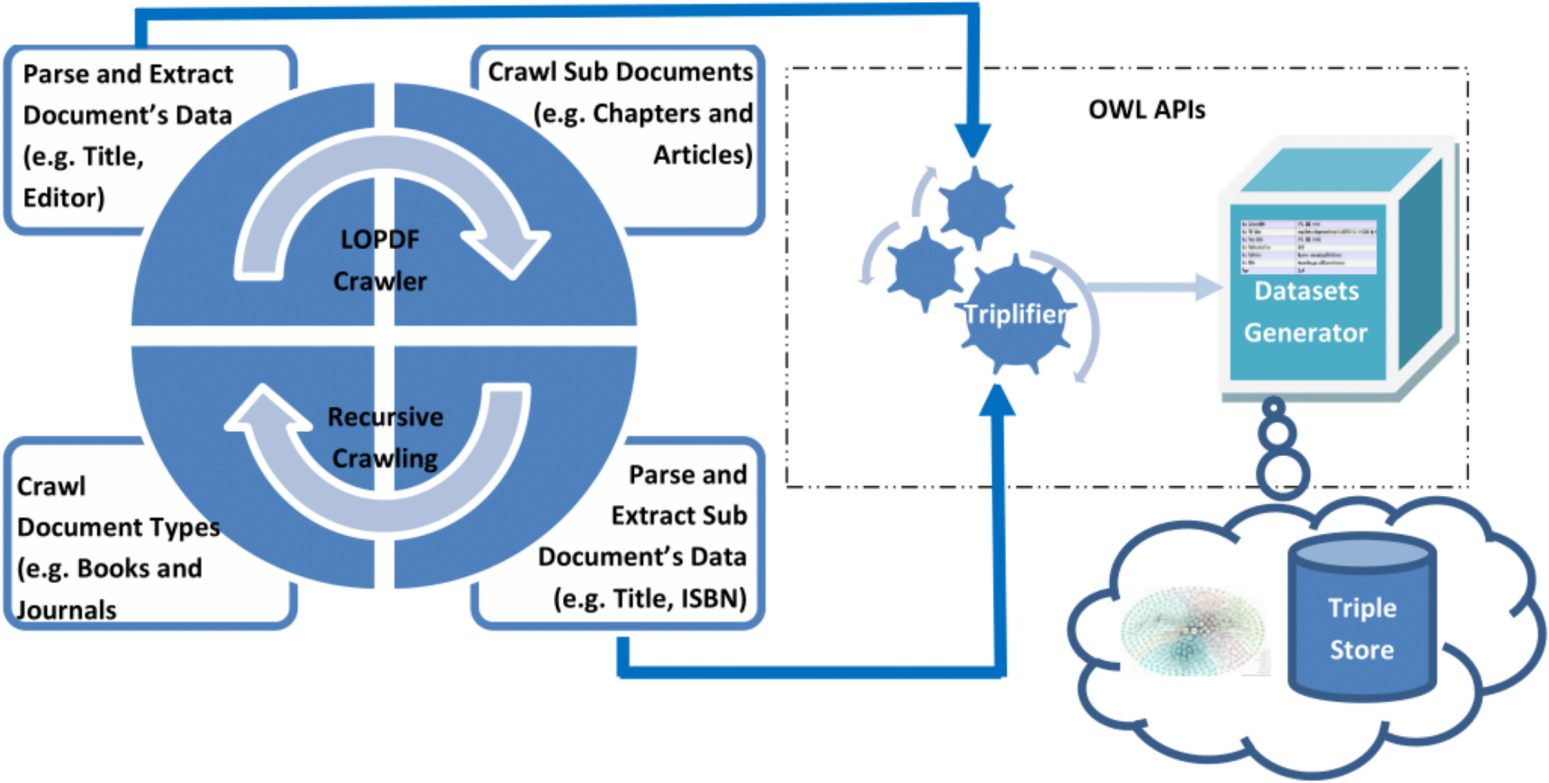
Architecture of the LOPDF framework.

Data about scientific publications is available on the Web as well as on publishers Web sites/ portals/ XML exports expressed by using standard terms (e.g., *title, publisher, author, ISBN* etc.). Based on study of different sources of scientific publications, we came up with two key challenges in extracting the LOD from existing sources: first challenge was to crawl the source information in such a way that none of the entity is left unprocessed and relational information (i.e., links) between different entities are not missed, second challenge was to parse and extract correct information from the information templates. Considering these challenges, *LOPDF* framework architecture is designed to resolve them with flexibility of customization. *LOPDF* framework architecture consists of four main modules: (i) *Information Crawler* (ii) *Information Parser and Extractor* (iii) *Triplifier* (iv) *Datasets Generator* (as shown in Fig. 3). The architecture of LOPDF framework is designed in such a way that LOPDF extraction process execute these modules in sequence by using a recursive approach (as shown in Fig. 3). Every module of the extraction process actually completes a specif part of the overall job and let the other modules to do their jobs and in this way all these four modules continue to execute in sequence recursively. In this section we briefly describe the LOPDF architecture and data extraction process that we used to extract semantically enriched data from plane and semi-structured text available in information specific templates of publisher specific sources.

**Figure 4:**
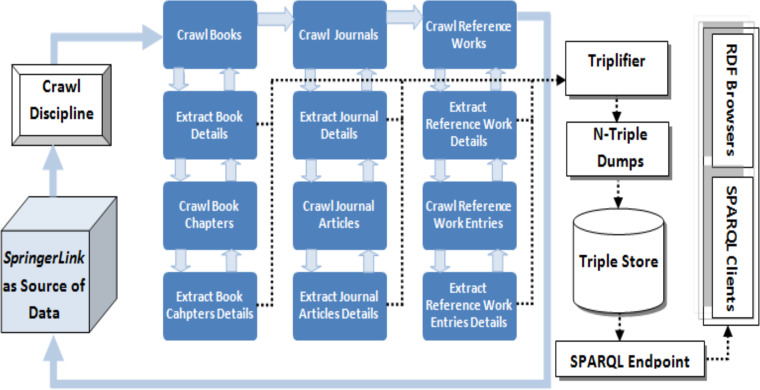
Data extraction process of the LOPDF framework (Aslam & Aljohani, 2016). Reprinted by permission from Springer Nature: Springer; Web-Age Information Management: 17th International Conference, WAIM 2016, Nanchang, China, June 3-5, 2016, Proceedings, Part I; Spedia: A semantics based repository of scientific publications data; Aslam MA and Aljohani NR; Copyright 2016.

***Information Crawler:*** Information crawling is the first phase of the information extraction process which takes the URL of the source portal as an input and starts crawling it from the first upto the last discipline. The information crawling process takes place at three levels i.e., (i) discipline level, (ii) parent document level and (iii) child document level (as shown in Fig. 4). Crawler starts crawling from the first discipline (e.g., *Architecture and Design*), then crawls every parent document (e.g., *book, journal* and *reference work*) in every discipline and then every child document (e.g., *chapter, article* and *reference work entry*) as child of every parent document. As an example we can consider the crawling process to be starting from *Architecture and Design* discipline, then it crawls across all *books* in this discipline and then every *chapter* of every book and so on.

***Data Parser and Extractor:*** Data parsing and extraction takes place at every stage of information crawling phase. Even information crawling phase could not be completed without execution of data parser and extractor module. The reason is that crawling phase needs the information about parent and child documents pages to be parsed, extracted and fed as input for the crawler to crawl to the next source of information. At the same time in this phase required information is extracted i.e., relations/links between parent/child documents as well as metadata about parent and child documents is parsed and extracted. As an example, in this module the parsing and extraction processes applies first to the book entity, then to the metadata of every constituting page, and finally to the metadata of chapter entities. This process generates all the metadata of parent and child documents as well as relational information pertaining to parent–child documents and between parent–child documetns and other entities such *author* and *editor*.

***Triplifier:*** Every output of the information parsing and extraction module is fed as input for the triplification module. In this phase every piece of data is processed so that it could be triplified, URI issues are addressed and inconsistencies in the extracted data are resolved. After purifying the extracted data, it is triplified and stored in the relevant data model. Information about every resource such as *book, chapter, journal, article, reference work, reference work entry, author, editor, editor-in-chief*, is triplified as resources. Properties for every resource are identified and mapped to relevant values (either as other resources or as literal values). Data values of some data type properties are not mapped to the true unit of the value; for instance, the publication date for some journal articles in the source data is mentioned as date, and for some as string (we further elaborate on such inconsistencies and obstacles in coming Section). All triples are stored in data models that are forwarded to the dataset generator module to produce the final datasets.

***RDF Datasets Generator:*** Data models that are created during the triplification module are taken as processing source in this phase. In fact data models are created for different resources and properties of these resources which are finally executed to generate resulting RDF datasets in N-Triple format. These datasets are loaded into the triple store server and can be used for asking smart questions by making use of SPARQL protocols or to browse by using semantic Web browsers to crawl across the linked data.

## LOPDF Data Extraction Algorithm

*LOPDF* data extraction algorithm uses a recursive approach to process and triplify the source data. The extraction algorithm is based on a general approach and can be customized with the publisher specific data sources. In the context of this paper it is customized for *SpringerLink* (as a source of data). The data parsing and extraction algorithm consists of two sub-algorithms, one is used to crawl and second is used to parse and extract required data. Both algorithm work in sequence and are dependent on each other.

The crawler algorithm takes the link of the source portal as an input and results in semantically enriched datasets in N-Triple format (as shown in Algorithm 1). Recursion plays a key role in crawling algorithm by crawling between disciplines, parent documents (e.g., books, journals, reference works) and then child documents (e.g., chapters, articles, reference work entries). At every stage of crawling step, parser algorithm is used to parse and extract metadata information as well as links of parent ∖ child documents that are used as input for the crawler. Whenever, crawling algorithm reaches a milestone which contains metadata or link to next source of information, the control is shifted to data parsing and extraction algorithm (as shown in lines 10, 13 of Algorithm 1). Also, the union operation in lines 10 and 13 of Algorithm 1 with RDF triples shows that RDF triples that are generated for the extracted data at this stage are unioned with the triples generated for the data extracted about the child documents. The triples output about child document is represented in line 26 of Algorithm 2, that’s why the lines 10, 13 of, Algorithm 1 are unioned with Algorithm 2.

Data parsing and extraction algorithm takes the URL of a document page as input and results in data models of the information extracted from the input URL. This algorithm parse the whole page at a given URL and extracts data in two categories, one is metadata of parent ∖child documents such as *title, abstract, isbn, doi* and second is links (URLs) to next source of information such as links of all chapters of a book or links of all articles of a journal (as showing in Algorithm 2). Every piece of metadata is extracted by parsing the available text, triplifing it and storing in the relevant data model. After parsing, extracting, triplfying the required data, control is transferred back to the crawler so that it can crawl to next source of information which ultimately again can be used as input for data parsing and extraction algorithm to extract required data and triplify it. In this way recursion plays an important role in processing each and every data item in the source portal and in extracting the parent child relations between documents and links between other data entities.

## Analysis of Extracted RDF Datasets

As discussed above that the architecture and data extraction process of LOPDF framework is designed in such a way that it could be customized by doing small changes in the end point triggers of the framework based on the structure and templates of the data source. We have already customized and applied the LOPDF framework on the *SpringerLink* as source of data and have created a knowledge base (named as *SPedia* (Aslam & Aljohani, 2017; Aslam & Aljohani, 2016)) of semantically enriched data about scientific publications published by *Springer*. In this section we give short introduction of SPedia as a product of LOPDF framework and provide the quantitative as well as qualitative analysis of datasets produced by using our framework.

**Figure 5:**
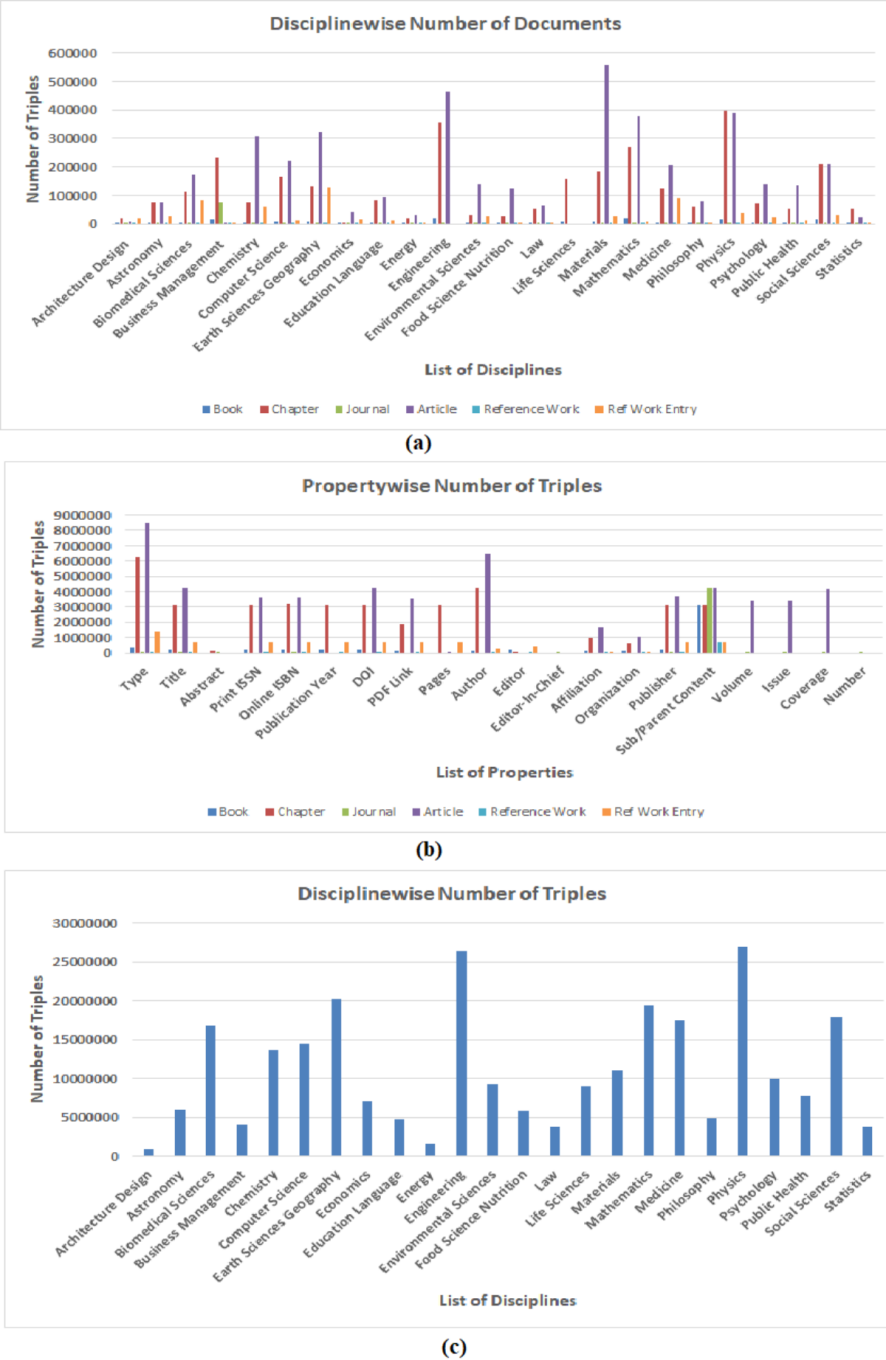
Statistical graph of (A) number of documents processed in every discipline grouped by document type (B) number triples extracted for every property grouped in all discipline (C) number of triples extracted in every discipline.

### SPedia knowledge base as product of LOPDF framework

*SPedia* is semantically enriched knowledge base of scientific publications data that we have extracted by using LOPDF framework. It consists of around three hundred million RDF triples describing information on about nine million scientific documents in machine processable format. Datasets of these three hundred million RDF triples provide metadata as well as relational information between different resources such as relation of an article to journal, author, organization and metadata of documents. There are some standards terms that are being used among all publishers to present metadata of scientific documents. Some of these common terms include *isbn, abstract, doi, author, title, editor* etc. These terms are organized in different templates by different publishers for the sack of metadata organization and management. We used these pre-defined terms as keywords, extracted the metadata related to them and mapped them to LOPDF ontology classes and properties. This mapping resulted into the publications data in RDF format where each data item is triplified in the *subject, predicate* and *object* format. Table 1 shows some sample RDF statements that are created from the metadata which is extracted from the data source by using LOPDF framework.

**Table 1:** Sample RDF statements generated by LOPDF framework, showing the metadata of a journal.

**Subject**	**Predicate**	**Object**
spedia:Journal_of_Cryptology	spedia:has_Title	”Journal of Cryptology”.
spedia:Journal_of_Cryptology	rdf:type	”Journal”.
spedia:Journal_of_Cryptology	spedia:has_Journal_No	”145”.
spedia:Journal_of_Cryptology	spedia:has_Online_ISSN	”1432-1378”.
spedia:Journal_of_Cryptology	spedia:has_Editor_In_Chief	”Kenneth G. Paterson”.

### Quantitative analysis of extracted datasets

Since, RDF datasets generated by using LOPDF framework consists of around three hundred million RDF triples (which are quite big datasets) and these datasets provide information about different document types grouped in different disciplines, therefore, providing the quantitative analysis of extracted number of resource as well as number of triples can be helpful in evaluating and using semantically enriched data of scientific publications. Scientific documents (in source and resulting RDF datasets) are categorized as *books, chapters, articles, journals, reference works* and *reference work entry* which are further categorized/grouped in disciplines such as *architecture design, engineering, computer science*. Figure 5 shows the statistical graphs of number of resources and triples extracted at different levels. Figure 5A shows the statistical graph of number of resources/documents grouped in disciplines i.e., number of resources extracted for *book, chapter, journal, article, reference work* and *reference work entry* grouped in disciplines such *architecture design, astronomy, biomedical science*. Figure 5A also shows that highest ratio of extracted resources and triples belongs to *earth sciences, engineering* and *physics* disciplines.

Figure 5B shows the statistical graph of number of triples extracted for some common metadata entities as properties. It shows very low ratio of information (i.e., number of triples) extracted for properties such as “*Abstract, Editor_In_Chief*” and “*Number*”. The reason of low percentage of these properties is that they belong to *Journal* which is second lowest number resource in the source portal (i.e., 3100). Also, the high percentage of “*Type*” and “*Sub/Parent Content*” properties is due to the reasons that every document has two types i.e., (i) document type such as *book, chapter, article* and (ii) discipline type such as *computer science, engineering*, and every document has its sub or parent document such as *book* has *chapter* and *chapter* belongs to *book*.

Finally, Fig. 5C shows statistics of total number of triples generated by using LOPDF framework in every discipline. Figure 5C also shows that highest number of triples are extracted from *engineering* and *physics* disciplines. Reasons of varying disciplines between Figs. 5A and 5C is that the overall number of documents processed in *engineering* and *physics* disciplines are more than overall number documents processed in *earth sciences* discipline and this over all number of documents belonging to these disciplines resulted in higher number of triples generated in these disciplines. This is also a way to analyze the quantity of extracted datasets.

### Qualitative analysis of resulting RDF data

Quality of RDF datasets produced by using LOPDF framework was tested and analyzed by a team of ten researchers having expertise in fields of linked open data, semantic Web and SPARQL query writing. As a part of qualitative analysis, extracted datasets were first loaded in the triple store server (making it one knowledge base) and SPARQL Endpoint was established. Every member of the testing team performed around thousand SPARQL queries (in average) to test and analyze results from different aspects including the queries for required documents by specifying different properties such as *title, author, editor, date* and complex queries such as finding documents based on interlinked information and metadata.

**Figure 6:**
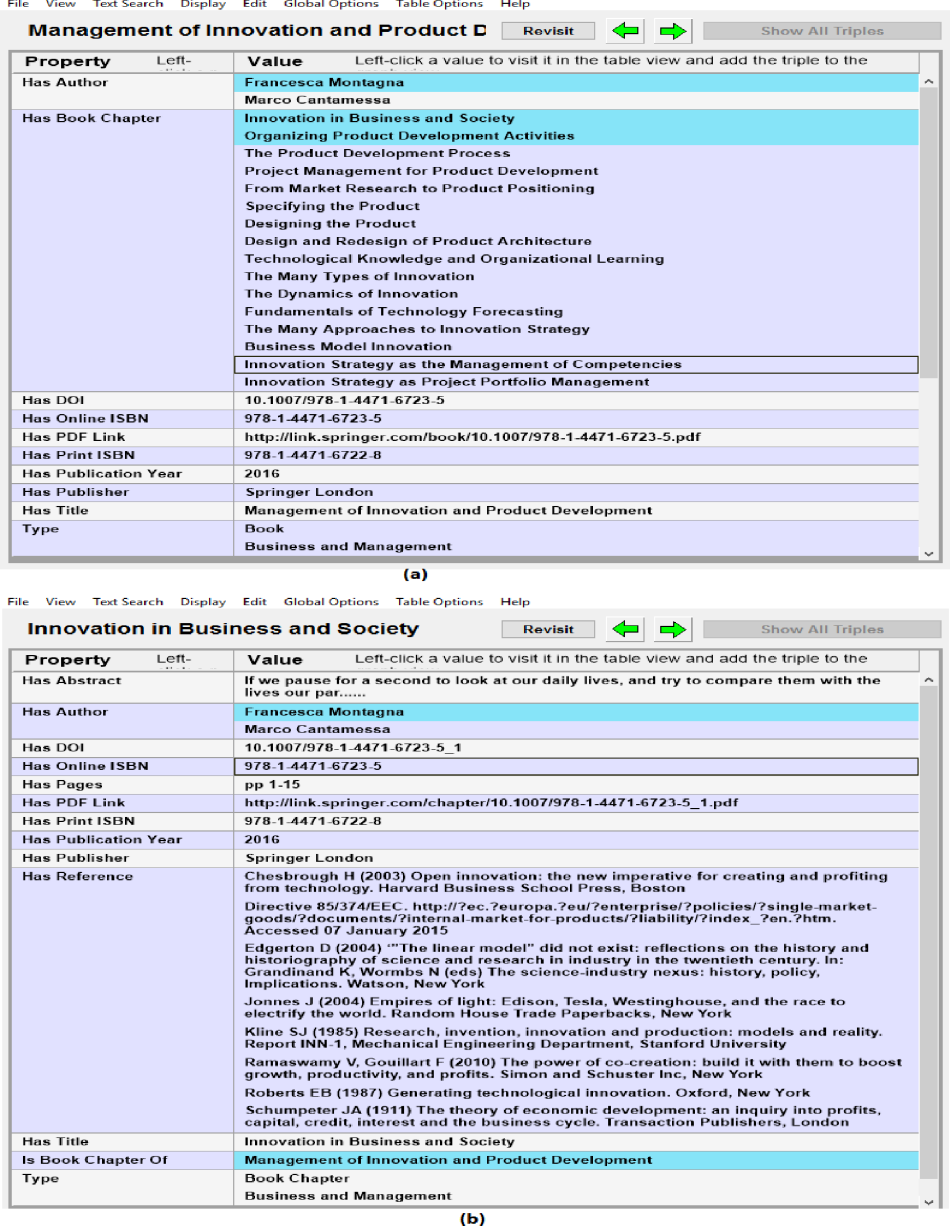
Analyzing quality of extracted data in semantic Web browser (i.e., Gruff) (A) showing metadata and links of book with child book chapters, and (B) showing book chapter metadata and its links to authors and parent book.

Metadata of documents from RDF datasets was also verified by connecting the SPARQL Endpoint with client semantic web browser (e.g., Gruff) and browsing the data in visual and tabular environment and verifying documents metadata with source portal. Gruff is the well-known semantic web browser that can be used to load the RDF data in it or to connect it with the SPARQL Endpoint and then to browser the data in visual form or in tabular form. It can also be used to make SPARQL queries to the connected SPARQL Endpoint and then analyzing the results in the visual environment of Gruff. Figure 6 shows sample snapshots of browsing the RDF datasets in semantic web browser. Figure 6A shows metadata such as authors, book chapters, doi and ISBN of the book and Fig. 6B shows next stage of browsing i.e., moving from book to the 1st chapter of the book by using property has_Book_Chapter. Figure 6B also shows the metadata of the book chapter that was verified against source data. In this way quality and accuracy of extracted metadata and links between different resources was assessed and verified.

**Figure 7:**
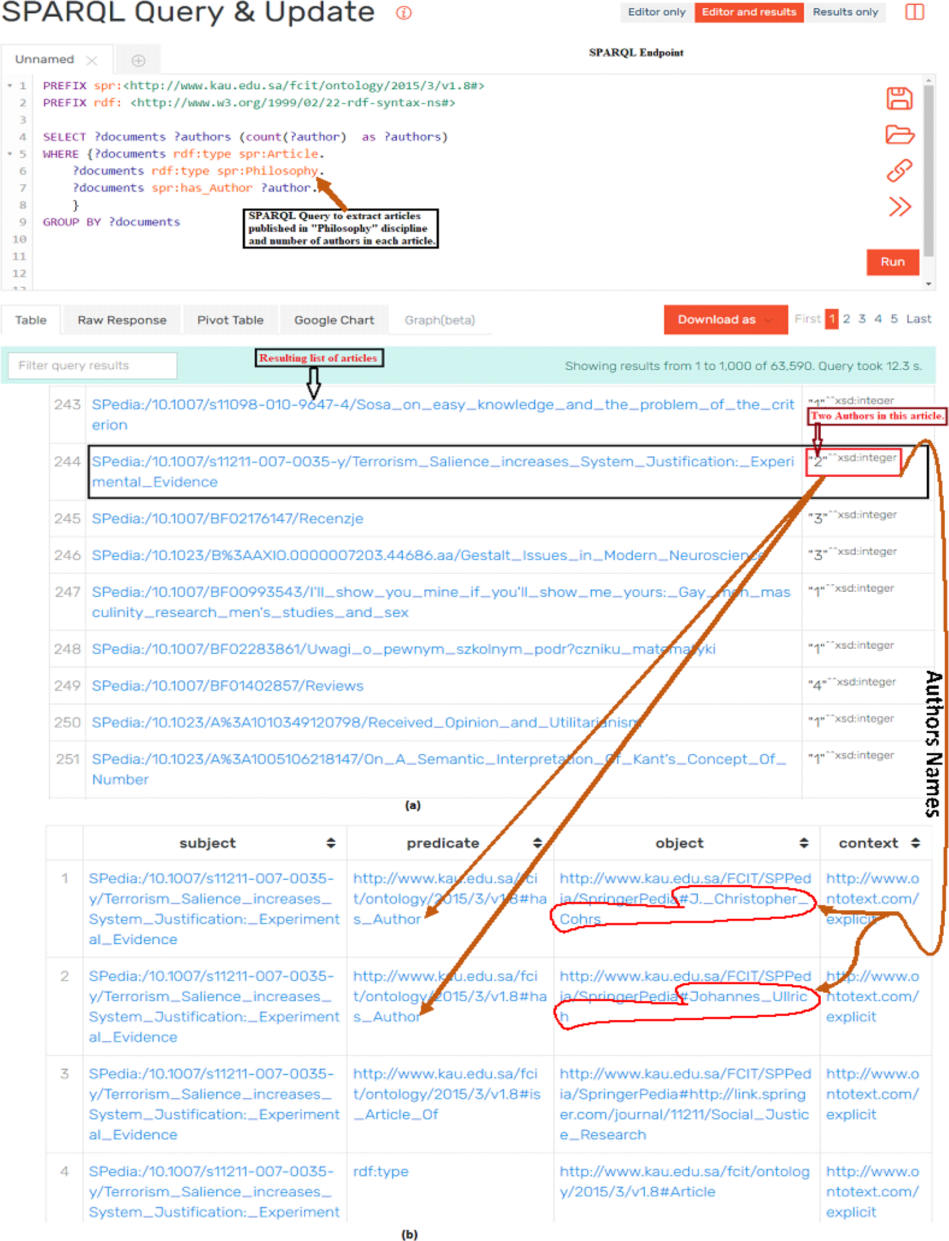
Assessing the quality of data by executing SPARQL query and analyzing the results of query: (A) The SPARQL query which resulted in articles published in Philosophy and number of authors of each article; (B) detailed metadata (i.e., names of authors) of the article.

We also used SPARQL queries for analyzing the quality of extracted data. Figure 7 shows a scenario of qualitative analysis of extracted data by making use of SPARQL queries through SPARQL Endpoint. Figure 7A shows a SPARQL query to extract journal articles published in *Philosophy* and number of authors of every article. The accuracy of extracted data was verified by randomly choosing articles from the list of results of SPARQL query and verifying documents types through source portal. The second aspect of data validation was to verify the number and names of authors of articles. Figure 7B shows sample data of validating names and numbers of authors of a particular article from Fig. 7A. Also, Fig. 7A shows one article that is highlighted with black box having 2 number of authors (highlighted in red box). When we go in further detail of this article (as shown in Fig. 7B), we notice that this article has 2 authors and their names are also highlighted in red boundary. This extracted data is cross checked with source data to verify the accuracy and quality of extracted data. This process is repeated across the various disciplines and article of these disciplines. Similarly SPARQL queries to fetch the metadata such as title, doi, pdf link, and links between different entities such as authors, affiliation, homepage are also queried for different documents and in different disciplines. In this way quality of data was verified for all disciplines and types of documents by querying specific data and by cross validating the data between produced RDF datasets and source data (i.e., *SpringerLink portal*).

There are over 10 million scientific documents in the source portal that are categorized into 24 disciplines. In each discipline these documents are further categorized into 12 types but due their potential importance and availability of sufficient number of documents, we consider six types of documents (i.e., *book, book chapter, journal, article, reference and reference work entry*). We did the discipline wise and type wise analysis of documents and at the end we found that approximately 88% percent documents are extracted from the source portal. The reason of not getting success in crawling and extracting metadata of 100% of documents was due to buffering the documents HTML text by using HTML libraries and then parsing and executing sub-crawlers (we are working on improving the algorithm to crawl and process as equal number of documents as in the source data). In addition to that, not considering the other 6 types of documents is also a reason of not having the same number of documents in the SPedia as in the source portal. With respect to the metadata of scientific documents we found 100% accuracy in the metadata of documents and in inter linking different datasets. The reason of this good percentage of accuracy in extracted metadata is efficient handling of obstacles and inconsistencies during the crawling, extracting and RDF datasets generation (as discussed in the next Section). We are working on developing third party applications and mashups by extracting more publications data from other publisher specific resources and then interlinking all as cloud of linked open scientific publications data. Initially, the process of data crawling and extraction was quite time consuming but improved the performance of the system (i) by improving the data extraction algorithm (which is already described in data extraction Section and (ii) by performing our experiment in High Performance Computing Environment (HPC) (i.e., Aziz supercomputer https://hpc.kau.edu.sa/Default-611997-EN).

### Obstacles and handling inconsistencies in source data

In our first attempt we used the *SpringerLink* portal as an input for the LOPDF framework. Many issues and inconsistencies were noted in the source data during the crawling and extraction process. These inconsistencies in source data resulted in many obstacles for the data extraction process to parse and extract right data values for different properties. Some frequently faced issues in the source data are discussed below.

Publication date is one of the main metadata attributes of any scientific document which may help in better inter linking as well as searching of documents over linked structure. In the data source, publication dates of documents are some times coated in the *date* format and some times in a *string* format, which may lead to wrong data values in the resulting datasets. Such inconsistencies in the publication date are resolved by parsing the date values in a coherent way and mapping them to string values in resulting RDF triples.

Similarly, sometimes data values in source portal are not consistent with standard headings/terms. For example, in some pages, information about single *editor* is presented by using the heading ‘Editors’. This heading leads the parsing process to extract multiple editors names whereas actually there exists only one. Likewise, *ISBN* numbers, in the source portal are sometimes documented as *numbers* and some times as *strings* by adding special character (i.e., ‘-’). Such values of *ISBN* numbers could not be type casted to integer values. That’s why *ISBN* numbers are mapped to string values to resolve these issues. In addition to all above mentioned issues, an important aspect is to resolve inconsistencies in URIs. As discussed before that every document (e.g., *book, chapter, journal*) is identified as a resource in RDF datasets. To export every document as a resource, a URI is created by using the *title* of the document as part of URI. Sometimes, *titles* of documents contain characters such as language-based special characters, mathematical signs, scientific special characters, which can not be used as a part of URIs (further details about best practices for publishing RDF vocabularies can be found in Phipps (2008). Such kind of characters are categorized as illegal characters for creating URIs and are replaced with most appropriate options, following the best practices for creating URIs.

## Potential Usage of LOPDF Framework

LOPDF framework is a general framework that can be used to extract and produce smart data from different publisher specific sources by making small changes to the end point trigers of the framework based on publisher specific templates. The generalized architecture of the LOPDF framework make it easily usable to process and extract publications metadata from different publisher specific sources as RDF based open datasets and to interlink these datasets by using the concept and principles of publishing LOD (Bizer, Heath & Berners-Lee, 2009).

RDF datasets produced by using the LOPDF framework can be used to ask smart questions for different data analysis purposes which otherwise are not possible to be performed on textual data. For example, we can use the Linked Open Data of scientific publications to perform different kinds of co-author network analysis such as author contribution detection, author order patterns detection, community and sub-community detection and to find influential authors in particular research areas. It can also be use to find rising research areas and rising organizations in specific research areas based on the LOD of authors, organizations and key-words of research publications. For organizational policy making purposes, we can also use these datasets to find authors collaboration patterns, multi-author trends in different disciplines and documents and for anomaly detection based on linked open data of documents and references of these documents. Performing these kinds of queries such as finding multi-author trend in writing scientific documents in different disciplines may help the management to find out disciplines which needs to establish policies for more collaborative work.

## Conclusion and Future Work

Due to the potential usage, acquisition and processing of open data, it is getting more and more attention to publish the individuals as well as organizational data as open data. Different sectors such Government, Education, Health and Transport around the world are publishing their public data as open data, making it easy for public as well as scientific researchers and policy makers to compute the contribution of different sectors in the overall behavioral change in the growing knowledge society. On the other hand when it comes to smart libraries in smart cities, a very few work has been done in publishing the open data of scientific publications, even though it can help a lot in finding individuals as well as organizations working in similar area and domain of interest, which ultimately can help in establishing cooperation and means of joint work to improve the state of the art and to produce better output. One of the main reason of lacking behind in publishing open data of scientific documents for smart digital libraries is the need of such frameworks that can be used to crawl, process, extract and produce semantically enriched from different publisher specific sources.

To address this limitation, in this paper, we have presented a generic framework (named as Linked Open Publications Data Framework (LOPDF)). LOPDF framework can be used to crawl, process, extract and produce RDF datasets of scientific documents. We also presented the architecture of LOPDF framework and how its different components can be used to crawl and process the different data entities. We also described the recursive algorithm that we developed to process the source data in such a way that none of the metadata entitiy nor links between different entities are lost or left unprocessed. Detailed quantitative as well as qualitative analysis of extracted datasets is described in the paper. This analysis shows the quality as well as accuracy of the resulting datasets. A case study to prove the potential usage of resulting RDF datasets is also discussed in detail. The case study shows that how the semantically enriched data of scientific documents can be used to perform different types of analysis that otherwise is not possible to be performed on textual data.

As a part of future work, we are extending the coverage of LOPDF framework to different domains and sources to achieve the goal of smart data. In addition to this we have extended and continuously extending and implementing this framework in other domains such as government, education and social media data. That’s why we did not make the framework open source. We are also working to publishing more usage scenarios of these publications RDF datasets. It will produce more confidence to the published work and to the LOD community in producing and using semantically enriched data.

## Acknowledgements

The authors acknowledge with thanks the Deanship of Scientific Research’s technical support.

##  Supplemental Information

10.7717/peerj-cs.445/supp-1 Supplemental Information 1 Sample Datasets and Framework Source Code.

## Additional Information and Declarations

### Data Availability

The following information was supplied regarding data availability: Data and are available in the Supplemental Files.
